# Simultaneous Use of Continuous Glucose Monitoring (CGM) Systems and the Remote Electrical Neuromodulation (REN) Wearable for Patients with Comorbid Diabetes and Migraine: An Interventional Single-Arm Compatibility Study

**DOI:** 10.3390/jcm15031097

**Published:** 2026-01-30

**Authors:** Yara Asmar, Alit Stark-Inbar, Maria Carmen Wilson, Katherine Podraza, Christina Treppendahl, Cem Demirci, Richelle deMayo

**Affiliations:** 1 Theranica, Netanya 4250438, Israel; 2 Department of Neurology, Ochsner Health, Covington, LA 70433, USA; 3 Ayer Neuroscience Institute Headache Center, Hartford HealthCare Medical Group, Mystic, CT 06355, USA; 4 Department of Neurology, University of Connecticut School of Medicine, Farmington, CT 06030, USA; 5 Ola Migraine Inc., Honolulu, HI 96814, USA; 6 Theranica, Bridgewater, NJ 08807, USA; 7 Pediatric Endocrinology and Diabetes, Connecticut Children’s, Hartford, CT 06106, USA; 8 Pediatric Endocrinology, Connecticut Children’s Medical Center, University of Connecticut School of Medicine, Farmington, CT 06032, USA; 9 Pediatric Headache Center, Connecticut Children’s Specialty Care Center—Westport, Westport, CT 06880, USA; 10 Division of Pediatric Pain Medicine, University of Connecticut School of Medicine, Hartford, CT 06061, USA

**Keywords:** migraine, diabetes mellitus, remote electrical neuromodulation, continuous glucose monitoring, wearable medical devices

## Abstract

**Background/Objectives:** Migraine and diabetes mellitus are highly prevalent chronic diseases, and their comorbidity presents management challenges, particularly when wearable medical technologies are used concurrently. Remote electrical neuromodulation (REN; Nerivio^®^) is an FDA-cleared non-pharmacological migraine therapy, and continuous glucose monitoring (CGM) systems are widely used in diabetes care. However, the safety and compatibility of simultaneous co-use have not yet been evaluated. This technical compatibility study aimed to assess whether REN operation affects CGM performance or interferes with glucose measurement integrity in diabetic adults. **Methods**: Twenty-one adults with diabetes using Dexcom G6/G7 or FreeStyle Libre 2/3 participated in a single-arm interventional study. During a 45 min session, participants operated the REN and CGM devices simultaneously on their smartphones, and the REN device was paused three times to compare CGM readings between REN ON and RED OFF conditions. The primary outcome was the mean absolute relative difference (MARD*_REN ON/OFF_*), evaluated against a prespecified 5% threshold. Statistical analysis included the Wilcoxon test, with subgroup analysis by the CGM device family. **Results:** The median MARD*_REN ON/OFF_* across all participants was 1.61% (IQR 0.84–2.44%), significantly below the 5% threshold (*p* < 0.001). All participants achieved MARD*_REN ON/OFF_* < 5%. Subgroup analyses were consistent: the median MARD*_REN ON/OFF_* was 1.70% (IQR 0.90–2.45%) for Dexcom and 1.05% (IQR 0.83–1.50%) for Abbott. No technical interference, Bluetooth disruptions, missed data transmission, or adverse events were observed. **Conclusions:** Simultaneous use of Nerivio^®^ REN and CGM systems in adults with diabetes is compatible and safe, with no evidence of interference or significant deviations in glucose readings. These findings support the integrated and reliable use of REN and CGM wearables in adults with diabetes managing comorbid conditions.

## 1. Introduction

Diabetes affects approximately eight hundred million people worldwide. Most of them have type 2 diabetes, and approximately 9.2 million people live with type 1 diabetes, including about 1.8 million youth under 20 years. Migraine affects over one billion people worldwide. Both diseases are major contributors to global disability [1,2,3]. The epidemiologic prevalence of migraine among individuals with diabetes is generally similar to that in people without diabetes, though it varies across studies. In the USA, an estimated 4.2–4.6 million individuals have co-occurring migraine and diabetes. Individuals with these comorbid diseases often experience lower quality of life, greater health care utilization, and higher medical costs than those with either condition alone, underscoring the need for integrated strategies to minimize management conflicts and optimize outcomes [4,5].

Although migraine and diabetes have distinct pathophysiological heterogeneous causes, several studies suggest they may share overlapping mechanisms—particularly in type 2 diabetes—including insulin resistance, systemic inflammation, endothelial dysfunction, and oxidative or mitochondrial stress. These shared pathways may help explain their observed comorbidity [6,7]. Clinically, migraine attacks may affect glycemic control by disrupting sleep, meals, and medication adherence. Conversely, diabetes-related dehydration, hyperglycemia, and hypoglycemia may trigger or exacerbate migraine [6]. Because migraine has a complex, multisystem pathophysiology, treatment selection can be challenging, especially in patients with comorbid conditions [7]. Effective migraine management often combines acute and preventive strategies. However, pharmacologic treatment in patients with diabetes may be limited by metabolic or cardiovascular considerations [6,8]. For example, triptans may increase insulin secretion via serotonergic pathways and are contraindicated in patients with vascular diseases due to their vasoconstrictive effects. Traditional migraine preventives such as tricyclic antidepressants are associated with weight gain, beta blockers can mask hypoglycemic symptoms and delay glucose recovery, and antiepileptic agents can alter acid–base and metabolic homeostasis [8]. Newer therapies, such as calcitonin gene-related peptide (CGRP) antagonists, also raise concern because CGRP shares structural similarity with amylin and may thus influence insulin secretion and energy expenditure as shown in experimental models, although the clinical relevance of these effects remains uncertain [6,7]. Therefore, patients with comorbid diabetes and migraine often search for drug-free migraine treatments.

FDA-cleared wearable device-based interventions have transformed management of both diabetes and migraine [9]. Continuous glucose monitoring (CGM) systems continuously measure interstitial glucose concentrations via subcutaneous sensors, transmitting data wirelessly to smartphone apps. CGM adoption has accelerated in recent years, especially following the American Diabetes Association’s (ADA) 2022 guideline update recommending CGM for all adults using intensive insulin therapy, supported by evidence that CGM improves glycemic control and reduces hypoglycemia [9,10]. Emerging glucose-sensing platforms such as microneedle and patch-based electrochemical systems reflect a broader shift toward minimally invasive, continuous monitoring. However, these platforms are not yet commercially established at the scale of current CGM systems [11]. Given the reliance of existing CGM systems on Bluetooth Low-Energy (BLE) communication and electrochemical signal detection, there is a theoretical risk of interference when other wearables emit electrical stimulation or BLE signals during co-use. This theoretical risk warrants empirical evaluation.

The remote electrical neuromodulation (REN) wearable is a drug-free, non-invasive device, FDA-cleared for acute and preventive migraine treatment in people aged 8 years old and above. REN is controlled by a smartphone application and worn on the upper arm during 45 min sessions. The device delivers electrical stimulation that activates descending pain inhibition pathways through a conditioned pain modulation (CPM) mechanism [12,13]. The REN device delivers localized biphasic electrical stimulation to the upper arm at low frequencies (approximately 100–120 Hz) via surface electrodes during discrete treatment sessions. This stimulation targets superficial tissues and does not extend into the subcutaneous interstitial space, where CGM sensors operate using amperometric electrochemical sensing that detects glucose through enzyme-mediated electrical currents. Therefore, direct interference with CGM signal generation is unlikely. However, indirect interference could theoretically occur through electromagnetic emissions from the stimulation circuity or through disruption of BLE-based wireless communication, as both devices operate in the 2.4 GHz band [12,13]. These possibilities underscore the importance of empirically assessing technical compatibility during simultaneous use.

Wearable solutions reflect a shift toward personalized, patient-centered, technology-driven management of chronic diseases. Both types of devices share key characteristics, including patient locus of control, smartphone integration, and continuous access to health data. Yet despite their growing use, the simultaneous co-application and safety of REN and CGM devices have not been systematically investigated, representing an important gap for people with complex comorbidity [14,15]. As people living with both conditions may rely on multiple wearable and app-based technologies in parallel, it is essential to confirm that REN and CGM devices can be used together without technical interference. Formal, structured compatibility testing of concurrent device operation is therefore an important component of digital health safety in multi-device use.

This technical compatibility study addresses the gap by assessing the safety and compatibility of simultaneous use of the REN wearable and commercially available CGMs in the USA. We hypothesized that REN use would not interfere with CGM function when both devices were operated simultaneously on the same smartphone.

## 2. Materials and Methods

### 2.1. Study Design

An interventional, single-arm, within-subject, repeated-measures, single-session technical compatibility study was conducted to assess potential device–device interference between simultaneous operation of the Nerivio^®^ REN wearable with commercially available CGM systems in the USA. The study aimed to determine whether simultaneous operation of CGM and REN devices via participants’ personal smartphones would result in changes in the readings of interstitial glucose levels or in any technical interference such as BLE conflict, connectivity interruptions, or impaired performance of either application.

### 2.2. Participants and Recruitment

USA-based adults who were actively using a CGM device for managing their diabetes were recruited through a moderated digital usability tool offering a video-based human insight platform (UserTesting Inc., Bellevue, WA, USA) and through social media campaigns. During the initial screening flow, candidates reviewed an electronic pre-consent statement and provided basic eligibility information. Candidates who met the initial criteria were invited to a follow-up virtual meeting, during which eligibility was fully assessed and confirmed by a study sponsor representative.

Eligible participants met the following criteria: (1) age ≥ 18 years; (2) living with diabetes; (3) actively using a CGM device connected to their personal smartphone and worn on their upper arm, abdomen, or other approved body locations, depending on device model and patient preference; (4) willingness to install the Nerivio^®^ app on their own smartphone—the same one used to operate the CGM app; and (5) at least 24 h of continuous CGM sensor wear prior to the in-person visit, with at least 8 h remaining for data recording at the time of the in-person visit. Migraine diagnosis was not a criterion for enrollment.

The study took place between 8 September 2025–18 September 2025. All participants provided written informed consent at the beginning of the in-person visit. The study protocol was reviewed and approved by Western Institutional Review Board (WIRB; 20252702). Participants were compensated for their time. All participant data were de-identified prior to analysis, and confidentiality was maintained in accordance with applicable data protection regulations.

### 2.3. Sample Size

A sample size of 20 participants was determined according to the U.S. Food and Drug Administration (FDA) human factors and usability engineering guidance, which recommends a minimum of 15 participants per group for validation-oriented studies focused on use-related safety [16]. As the study objective was to evaluate co-use safety and technical compatibility rather than clinical efficacy, no formal power calculation was performed. Building on prior usability studies conducted by Theranica, which followed the same FDA human factor framework, the chosen sample size was deemed sufficient to observe potential use errors and interactions relevant to safety for intended CGM users and the intended use environment. To achieve a final sample size of 20 participants, 25 candidates were invited to the in-person visit, accounting for a 20% no-show rate.

### 2.4. Study Devices

CGM systems including FreeStyle Libre 2 or 3 systems (Abbott Diabetes Care, Alameda, CA, USA) and Dexcom G6 or G7 systems (Dexcom Inc., San Diego, CA, USA), continuously measure interstitial glucose concentrations via subcutaneous sensors, transmitting data wirelessly to smartphone applications or to designated receiver devices. The dedicated smartphone application displays the user’s interstitial glucose level, which lags 5–15 min behind the blood glucose (BG) levels, as well as displaying trends and alerts for high or low glucose levels. The on-body and app-based design allows users to monitor glucose continuously and conveniently [17,18].

The remote electrical neuromodulation (REN) wearable (Nerivio^®^ by Theranica, Bridgewater, NJ, USA) is a drug-free, non-invasive device, FDA-cleared and CE-marked for both acute and preventive migraine treatment in people aged 8 years old and above. It is controlled (initiation, pause, and stimulation intensity) by a dedicated smartphone application (Nerivio^®^) and worn on the upper arm for 45 min acute or prevention treatments [12,13].

### 2.5. Study Procedures

Each participant completed a single session. At the beginning of the session, the REN application was installed on the participant’s smartphone, in addition to the CGM application that was already installed and continuously running. During the session, the REN wearable device ran for 45 min, and its operation was paused at three predefined intervals set by an independent stopwatch. The CGM system remained continuously active before, throughout, and after the session. Both apps operated on the smartphone system clock; therefore, timestamps across apps were inherently synchronized at the smartphone level.

**Figure 1:**
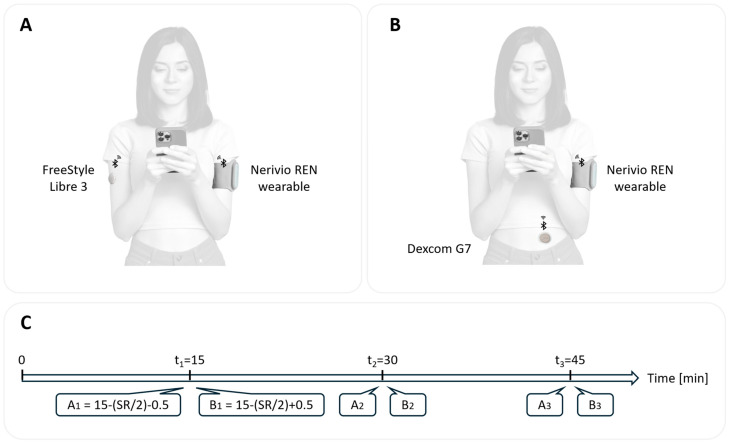
**Illustration of the study design and timeline.** (**A**) Illustration of a participant with a remote electrical neuromodulation (REN) wearable device and a FreeStyle Libre 3 continuous glucose monitoring (CGM) sensor, both using BLE to transmit data to the same smartphone. (**B**) Same, with a Dexcom G7 CGM sensor. (**C**) Study timeline, showing the sequence of REN activity and CGM data collection across the three timepoints (t_i_), A_i_ = REN ‘ON’, Bi_2_ = REN’ OFF’, SR = CGM sensor sampling rate. Timing intervals between ON/OFF readings were set according to each CGM system’s SR (intervals of 2 min for Freestyle Libre and 6 min for Dexcom, given corresponding SR of 1 and 5 min).

In cases where participants had the CGM on their arm, the REN wearable was placed on the other arm; if the CGM was on their abdomen, the REN was placed on their left arm (Figure 1A,B). Participants were instructed to set the REN intensity, according to clinical guidance, to a level that was strong, well felt, but not painful.

Three predefined time points—15, 30, and 45 min after initiating REN treatment—were chosen to capture the full complete cycle of an REN treatment, sampling early, mid, and late phases of simultaneous device operation. During these timepoints, paired CGM readings were obtained: one during active REN device operation (“ON” phase) and one during a paused interval (“OFF” phase). The interval between each ON/OFF pair exceeded the CGM’s native sampling rate (SR) to ensure independent and refreshed CGM data acquisition (Figure 1C). For the FreeStyle Libre 2/3 systems, which record glucose values every 1 min, paired data were collected 2 min apart (i.e., at 14 and 16, 29 and 31, and 44 and 46 min). For the Dexcom G6/7 systems, which sample every 5 min, paired readings were collected 6 min apart (i.e., at 12 and 18, 27 and 33, and 42 and 48 min). Potential BLE interference was monitored by observing any loss of connectivity, interruptions, or error messages in either the CGM or REN applications. Any technical problems or issues were to be documented in study logs.

### 2.6. Outcome Measures

The outcome metric was the mean absolute relative difference (MARD), which is widely applied to assess the clinical accuracy of CGM systems and has been a standard metric in the field for over 15 years [19,20]. Traditionally, MARD represents the mean absolute difference between CGM measurements and a reference value, divided by the reference value, and expressed as a percentage (see formula):

$$MARD\;=\frac{1}{N}\sum_{i=1}^{N}\left|\frac{CGM_{i}-\;Reference_{i}}{Reference_{i}}\right|*\;100\%$$

In the assessment of CGM system clinical accuracy, MARD is calculated between a CGM device and BG values obtained from venue or capillary samples. This approach reflects the traditional use of MARD to quantify the deviation of CGM readings from the ‘gold-standard’ measurements of BG, as reported in numerous clinical studies and performance standards [19,21]. Beyond this MARD’s classical application, it has also been applied to evaluate the degree of agreement or potential interference between two CGM systems operating simultaneously, and more generally, to compare CGM readings obtained under different conditions, as long as one condition is explicitly defined as the reference [22]. In this study, we defined a dedicated metric, MARD*_REN ON/OFF_*, representing the mean of the absolute relative difference (ARD) between paired CGM glucose values obtained during the REN ON state and REN OFF state, thus reflecting both physiological variability and potential device interference. MARD*_REN ON/OFF_* was calculated at each corresponding timepoint as

$$ARD_{i}=\left|\frac{CGM_{REN_{ONi}}-CGM_{REN_{OFFi}}\;}{CGM_{REN_{OFFi}}}\right|\times 100\%$$

The overall MARD*_REN ON/OFF_* was then computed as

$$MARD_{REN\;ON/OFF}=\frac{1}{N}\sum_{i=1}^{N}ARD_{i}$$

where *N* is the total number of paired measurements.

In most CGM performance studies, a MARD threshold of <10% is recognized as indicative of good analytical accuracy. The FDA’s special control requirements for integrated CGM systems (iCGMs), outlined in 21 CFR 862.1355, define acceptable accuracy as at least 87% of sensor values falling within 20% of reference values (lower one-sided 95% confidence interval) [23]. Since this study aimed to detect even minor deviations and assess device-to-device technical compatibility, rather than establish classical CGM-BG accuracy, a stricter <5% threshold was prespecified. This value, approximately half of the conventional 10% MARD benchmark, provides a conservative margin to flag potential REN-related effects within established CGM performance limits. To assess possible interference from simultaneous REN use, CGM measurements during the REN OFF state were designated as the reference baseline and compared to those during the REN ON using the metric MARD*_REN ON/OFF_*_._

Primary Outcome: Group Median MARD*_REN ON/OFF_*. Median MARD*_REN ON/OFF_* (and interquartile range; IQR) across all participants, providing a group-level measure of functional compatibility between the REN device and the CGM system during simultaneous use. A group median MARD of 5% was selected as the threshold to indicate minimal deviation [21].

Secondary Outcome: Participant MARD*_REN ON/OFF_*. Proportion of participants with a MARD*_REN ON/OFF_* value below 5%, corresponding to minimal deviation from baseline CGM values, per participant.

Technical and Safety Outcomes: Technical assessment included BLE disconnections, device-related usability issues (sensor malfunction, transmitter failure, false alarms, missed data transmissions), and app malfunctions during REN operation across any of the CGM systems tested. Safety assessment included adverse events and device-related adverse events,. Adverse events were assessed by clinical observation and verbal reports from participants.

### 2.7. Analysis

MARD*_REN ON/OFF_* values were calculated for each participant from the three sampling pairs. The distribution of participant-level MARD*_REN ON/OFF_* values was first assessed using histogram visualization. Given the non-normal distribution, a one-sided Wilcoxon signed-rank test was applied to determine whether the median group MARD*_REN ON/OFF_* was significantly lower than the prespecified 5% threshold. The proportion of participants with MARD < 5% was summarized descriptively as counts and percentages.

An exploratory subgroup analysis was conducted for each CGM family (Dexcom G6/G7; Freestyle Libre 2/3), comparing median group MARD*_REN ON/OFF_* values against the 5% threshold using the Wilcoxon signed-rank test.

Analyses were conducted on de-identified datasets according to a predefined analysis plan; REN ON and OFF phases were derived programmatically from time stamps and used as an analysis label, without access to other participant-level identifiers.

All analyses were performed on complete cases, with no missing data. Results are reported as medians (IQR) for corresponding nonparametric tests and mean ± SD for descriptive statistics.

## 3. Results

### 3.1. Participants

A total of 21 consenting participants were enrolled in the study, with a mean age of 49.9 years (±SD 11.2; range 31–77 years). The cohort included 11 females (52%) and 10 males (48%). In total, 15 participants (71%) identified as Caucasians, 2 (10%) as Asians, 2 (10%) as Black or African American, and 2 (10%) as Hispanic/Latino. Twelve participants (57%) had type 2 diabetes, and 9 (43%) had type 1 diabetes. HbA1c values ranged between 5.4 and 7.9 with a mean of 6.6 (±SD 0.69; data obtained from 17 participants). CGM systems included Dexcom G7 (n = 13; 62%), Libre 3 (n = 5; 24%), Dexcom G6 (n = 2; 10%), and Libre 2 (n = 1; 5%). Most participants used an iOS smartphone (n = 14; 67%), while seven (33%) used Android smartphones. With respect to sensor wear duration at the time of enrolment, 11 participants (52%) wore their CGM sensors for less than 3 days, 7 (33%) for 3–6 days, 1 (5%) for 7–9 days, and 2 (10%) for 10–13 days. All participants had worn their CGM for at least 24 h prior to enrollment and had at least 8 h remaining for CGM usage, in accordance with the study eligibility criteria. Most sensors (n = 13; 62%) were placed on the upper arm, while eight participants (38%) wore the sensor on the abdomen, consistent with manufacturer-accepted body locations. Table 1 shows per-participant characteristics.

### 3.2. Primary Outcome: Median MARD_REN ON/OFF_ Across All Participants

**Figure 2:**
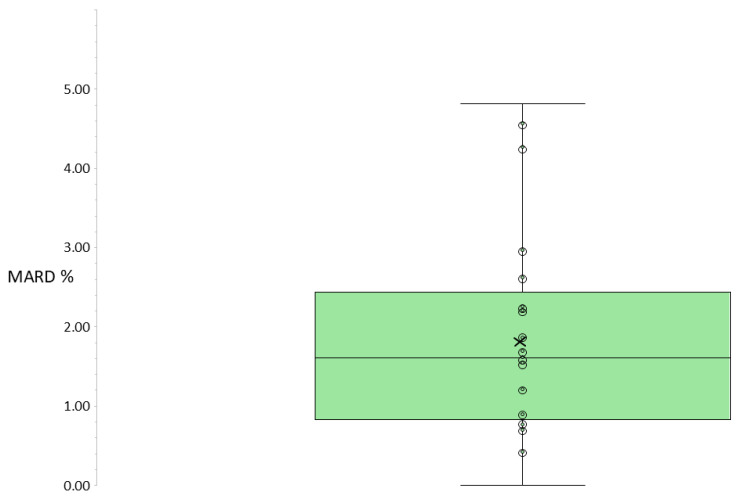
**Boxplot showing the distribution of MARD*_REN ON/OFF_* values across participants.** Each dot represents the MARD*_REN ON/OFF_* value for an individual participant. The “×” indicates the group mean. The box spans the interquartile range (IQR: 25th–75th percentile), and the line inside the box marks the group median MARD*_REN ON/OFF_*.

Across all participants and measurement points, mean glucose values ranged between 72 and 228 mg/dL (mean ± SD: 133.7 ± 39.2 mg/dL). Median MARD*_REN ON/OFF_* between “ON” and “OFF” readings of the REN wearable, i.e., with and without the active presence of REN, across all participants, was 1.61% (IQR: 0.84–2.44%), ranging from 0.00% to 4.81% (Table 2, Figure 2). The observed deviation was significantly below the prespecified 5% threshold (W = 0; sum of negative ranks = 231; sum of positive ranks = 0; z = −4.01; *p* < 0.001; one-sample Wilcoxon signed-rank test).

### 3.3. Secondary Outcome: Proportion of Participants with MARD_REN ON/OFF_ < 5%

The prespecified performance threshold of MARD*_REN ON/OFF_* below 5% was achieved by 21/21 participants (100%) (Table 2), indicating minimal deviation from baseline CGM readings across the cohort. This outcome is moreover substantially lower than the 10% threshold typically reported for CGM validation studies versus venous or capillary BG (blood glucose) measurements.

### 3.4. Exploratory Subgroup Analysis by Device Family

Exploratory analyses compared MARD*_REN ON/OFF_* values per device family. Among participants using Dexcom systems (G6 and G7; n = 15), the median MARD*_REN ON/OFF_* was 1.70% (IQR: 0.90–2.45%). A one-sample Wilcoxon signed-rank test demonstrated that the median MARD*_REN ON/OFF_* was significantly lower than 5% (W = 0; *p* < 0.001), confirming stable and consistent CGM performance during REN operation. Similarly, for participants using Abbott systems (Libre 2 and Libre 3; n = 6), the median MARD*_REN ON/OFF_* was 1.05% (IQR: 0.83–1.50%). The Wilcoxon signed-rank test showed a statistically significant difference below 5% (W = 0; *p* < 0.05), indicating that no functional interference occurred during simultaneous device use.

### 3.5. Technical and Safety Outcomes

There were no BLE disconnections, sensor malfunctions, transmitter failures, false alarms, missed data transmissions, or app malfunctions during REN operation across any of the CGM systems tested. Data collection logs indicated complete data capture for each ON/OFF measurement pair. No adverse events or unexpected CGM alarms were reported by participants.

## 4. Discussion

### 4.1. Key Findings

This technical compatibility study evaluated simultaneous use of the Nerivio^®^ remote electrical neuromodulation (REN) wearable with various continuous glucose monitoring (CGM) systems in adults with diabetes. The primary outcome demonstrated that the median MARD*_REN ON/OFF_* across all participants was significantly below the 5% threshold (median 1.61%, IQR 0.84–2.44%), confirming that REN activation did not interfere with the CGM signal performance. Importantly, all participants (21/21, 100%) achieved MARD*_REN ON/OFF_* < 5%, indicating minimal deviation in CGM readings during REN operation. Exploratory subgroup analysis, performed separately for each device family, showed that both Dexcom systems (G6 and G7; n = 15) and Abbott systems (Libre 2 and 3; n = 6) maintained MARD*_REN ON/OFF_* values significantly below 5% (all *p* < 0.05), supporting consistent device-to-device compatibility. No technical malfunctions, BLE interruptions, or adverse events were observed. Collectively, these findings confirm that the simultaneous use of REN and CGM systems is safe, feasible, functionally compatible, and does not compromise glucose data integrity.

### 4.2. Comparison with Previous Studies

Accuracy of CGM devices is typically assessed using MARD with a threshold of <10% widely recognized as analytically reliable for CGM-BG comparisons [24,25]. However, recent studies and methodological reviews emphasize that head-to-head device evaluations and advances in CGM technology can achieve, and in some cases warrant, a stricter threshold closer to 5%, to meet the standards of modern device validation and modeling. In this study, and in contrast to these classical applications, MARD*_REN ON/OFF_* was defined as a condition-specific metric that quantifies the relative difference between CGM readings during active and inactive REN states, serving as a measure of technical compatibility and potential interference rather than conventional absolute accuracy versus BG. Both the group-level median and participant-level MARD*_REN ON/OFF_* values remained well within the firm benchmark, underscoring the safe co-use of these two technologies. Although certain medications and substances have been associated with CGM sensor interference, this study provides new evidence that REN does not impact CGM accuracy or performance during real-world short-term use.

Several engineering studies have examined the wireless coexistence of BLE and other 2.4 GHz systems in body-centric and wearable applications, showing that well-designed BLE links can perform reliably even in congested radio frequency environments [26,27]. While these studies are technical, they suggest that BLE-based wearables can operate simultaneously without significant interference. Our findings extend this evidence, demonstrating that a BLE-smartphone-enabled REN device and smartphone-connected CGM system can function concurrently, on the same body and smartphone, without technical interference with app performance.

Prior work on wearable devices has shown that BLE-based devices can generally operate in parallel without significant interference [28,29]. Based on our knowledge, this is the first study to directly evaluate the use of REN and CGM sensors operating simultaneously on the same body and smartphone platform, thereby extending prior device accuracy and interference work into the context of co-used, BLE-enabled wearables in diabetes care.

### 4.3. Clinical Implications

The study results suggest that co-use of REN and CGM devices is unlikely to alter CGM derived glucose readings under common, stable conditions, supporting clinicians in prescribing both technologies for adults with diabetes who may also have migraine. Given the high prevalence and clinical impact of migraine in individuals with diabetes, the ability to combine continuous glucose monitoring with REN treatment offers a promising avenue for integrated symptom management.

Beyond the specific devices evaluated, this study illustrates a structured approach to compatibility and co-use safety testing for wearable medical technologies. As people living with comorbidities increasingly rely on multiple wearable and app-based tools in parallel, systematic evaluation of concurrent device operation is becoming an important component of digital health safety. Targeted compatibility assessments such as the MARD*_REN ON/OFF_* framework used here can help identify risks related to signal disruption, data loss, or misleading measurements. Such assessments can be performed either before broad deployment in clinical practice or as post-market surveillance studies to support safer multi-device digital health ecosystems for patients who depend on several technologies simultaneously.

In addition, this technical compatibility study is conceptually aligned with evolving FDA digital guidance and the European Medical Device Regulation (MDR), which emphasize that connected medical products should demonstrate reliable performance and safety in real-world multi-device environments [30]. As patients increase the co-use of multiple connected devices, compatibility testing should also serve as a regulatory and ethical expectation.

### 4.4. Strengths and Limitations

This study offers several strengths. It employed the first methodological real-time simultaneous evaluation of CGM glucose readings during REN stimulation across the most commonly used CGM sensor types in the U.S. The inclusion of the maximal number of measurement pairs (three) within a 45 min REN treatment session per participant, rather than a single measurement, minimized potential confounding effects of changes in glucose metabolism, adding to the strength of the study and focusing on direct device-to-device interaction.

However, several limitations should be acknowledged. First, MARD*_REN ON/OFF_* captures both physiological and technical variability. Measurements were conducted during relatively calm conditions without eating, exercise, sweating, or physiological stress, which affect glucose levels and may cause rapid glucose excursions. Thus, the physiological variability was expected to be quite low and stable during the session. Assessment of devices’ co-use under these conditions may therefore be warranted in future studies to further characterize compatibility across a broader range of real-world glycemic states. Correspondingly, glucose values measured during the session reflected that no one had hypoglycemia events, while some participants exhibited hyperglycemia. HbA1c values indicated a range from optimal to suboptimal (elevated) glycemic control, suggesting that the sample reflects a clinically relevant spectrum of diabetes rather than only well-controlled patients. The consistently low MARD values argue against clinically meaningful interference.

Second, adults with diabetes who do not have migraine were enrolled into the study, and thus device compatibility was not evaluated during migraine attacks. While assessing co-use during symptomatic migraine episodes and more diverse glucose conditions is an important direction for future research, our findings primarily characterize technical compatibility and safety under common real-world conditions, which were the goals of this study.

Lastly, the single-session single-arm design, and the relatively small sample size, may constrain the generalizability of the findings. Future studies with a randomized larger and more clinically diverse cohort may better reflect the populations most likely to use both devices. Specifically, the exploratory subgroup analysis for Abbott CGM systems included a limited number of participants (n = 6), with only one user of the Libre 2 system, which may constrain the generalizability of Abbott-specific findings. This distribution reflects real-world CGM utilization patterns in the United States, where Dexcom systems are more commonly used, particularly in regions such as the northeastern U.S. where the study was conducted. Accordingly, Abbott-specific results should be interpreted cautiously and warrant confirmation in larger, more balanced cohorts. Despite these limitations, the study consistently demonstrated that co-use of the CGM and REN devices did not produce interference or clinically meaningful deviations in CGM glucose measurements, effectively addressing the primary study goal.

## 5. Conclusions

These findings support the safety and compatibility of BLE-enabled wearable devices, CGM sensors, and the REN wearable in individuals managing both diabetes and migraine without disrupting continuous glucose monitoring. As digital health ecosystems expand, ensuring non-interference between devices will become essential for enabling people with comorbid conditions to safely benefit from drug-free treatments and connected solutions. This study provides technical feasibility and evidence supporting the potential for integrating the REN wearable therapy into real-world diabetes care for individuals living with both migraine and diabetes.

## Figures and Tables

**Table 1:** **Participant characteristics.** Demographics, continuous glucose monitor (CGM), and smartphone information per study participant.

Gender	Age	Race	CGM Type	CGM Location	Diabetes Type	Smartphone Type	Time Wearing CGM Sensor Prior to Trial	HbA1c Value
Male	66	Caucasian	Dexcom G6	Abdomen	Type 2	Android	3–6 days	7.1
Female	60	Caucasian	Dexcom G6	Upper arm	Type 2	iOS	3–6 days	5.4
Female	34	Hispanic, Latino	Dexcom G7	Abdomen	Type 2	Android	<3 days	N/A
Female	40	Black or African American	Dexcom G7	Upper arm	Type 2	Android	<3 days	5.6
Female	36	Black or African American	Dexcom G7	Abdomen	Type 2	Android	<3 days	6.9
Female	49	Caucasian	Dexcom G7	Upper arm	Type 1	Android	3–6 days	7.6
Female	51	Caucasian	Dexcom G7	Abdomen	Type 1	iOS	3–6 days	7.1
Male	58	Caucasian	Dexcom G7	Upper arm	Type 2	iOS	3–6 days	6.5
Male	31	Asian	Dexcom G7	Abdomen	Type 1	iOS	<3 days	7.5
Male	58	Caucasian	Dexcom G7	Upper arm	Type 2	iOS	<3 days	6.0
Female	53	Caucasian	Dexcom G7	Abdomen	Type 1	iOS	<3 days	6.4
Male	77	Caucasian	Dexcom G7	Upper arm	Type 1	iOS	<3 days	N/A
Female	42	Caucasian	Dexcom G7	Upper arm	Type 1	iOS	7–9 days	6.2
Female	47	Caucasian	Dexcom G7	Upper arm	Type 1	iOS	<3 days	6.2
Male	45	Caucasian	Dexcom G7	Abdomen	Type 1	iOS	<3 days	6.1
Female	56	Caucasian	FreeStyle Libre 2	Upper arm	Type 2	Android	<3 days	6.8
Male	47	Caucasian	FreeStyle Libre 3	Upper arm	Type 2	Android	<3 days	N/A
Male	42	Hispanic, Latino	FreeStyle Libre 3	Upper arm	Type 2	iOS	3–6 days	N/A
Male	53	Asian	FreeStyle Libre 3	Abdomen	Type 2	iOS	10–13 days	6.6
Male	60	Caucasian	FreeStyle Libre 3	Upper arm	Type 2	iOS	3–6 days	7.9
Female	43	Caucasian	FreeStyle Libre 3	Upper arm	Type 1	iOS	10–13 days	6.2

**Table 2:** **Participant’s Mean Absolute Relative Difference (MARD*_REN ON/OFF_*)**. Paired REN ON/OFF CGM readings (mg/dL), absolute relative difference (ARD; %) defined as the absolute percentage difference between the two readings per timepoint, and subject-level MARD (%) values. Participant order is the same as in Table 1.

CGM Type	A1 REN ON (mg/dL)	B1 REN OFF (mg/dL)	ARD1 (%)	A2 REN ON (mg/dL)	B2 REN OFF (mg/dL)	ARD2 (%)	A3 REN ON (mg/dL)	B3 REN OFF (mg/dL)	ARD3 (%)	MARD REN ON/OFF %
Dexcom G6	154	156	1.28	159	159	0.00	160	160	0.00	0.43
Dexcom G6	106	107	0.93	108	109	0.92	108	109	0.92	0.92
Dexcom G7	112	113	0.88	113	118	4.24	118	118	0.00	1.71
Dexcom G7	115	114	0.88	113	112	0.89	110	109	0.92	0.90
Dexcom G7	115	115	0.00	117	117	0.00	115	115	0.00	0.00
Dexcom G7	127	130	2.31	144	153	5.88	187	176	6.25	4.81
Dexcom G7	144	150	4.00	152	153	0.65	146	146	0.00	1.55
Dexcom G7	116	122	4.92	134	142	5.63	153	158	3.16	4.57
Dexcom G7	228	225	1.33	220	213	3.29	203	205	0.98	1.87
Dexcom G7	96	95	1.05	92	90	2.22	91	87	4.60	2.62
Dexcom G7	78	74	5.41	72	73	1.37	72	72	0.00	2.26
Dexcom G7	74	81	8.64	99	100	1.00	100	97	3.09	4.24
Dexcom G7	160	164	2.44	151	148	2.03	141	138	2.17	2.21
Dexcom G7	152	153	0.65	150	151	0.66	139	139	0.00	0.44
Dexcom G7	114	117	2.56	117	120	2.50	125	125	0.00	1.69
FreeStyle Libre 2	164	160	2.50	166	167	0.60	169	172	1.74	1.61
FreeStyle Libre 3	154	157	1.91	173	175	1.14	166	167	0.60	1.22
FreeStyle Libre 3	89	88	1.14	86	86	0.00	85	86	1.16	0.77
FreeStyle Libre 3	141	142	0.70	143	142	0.70	143	142	0.70	0.70
FreeStyle Libre 3	225	228	1.32	221	221	0.00	208	205	1.46	0.93
FreeStyle Libre 3	99	100	1.00	91	95	4.21	84	81	3.70	2.97
**Median**										1.61
**IQR**										0.84–2.44

## Data Availability

The data presented in this study are available on request from the corresponding author due to participant privacy and data protection considerations.
